# Theabrownin from Dark Tea Ameliorates Insulin Resistance via Attenuating Oxidative Stress and Modulating IRS-1/PI3K/Akt Pathway in HepG2 Cells

**DOI:** 10.3390/nu15183862

**Published:** 2023-09-05

**Authors:** Jia Liu, Xuan Wang, Yuanqin Zhu, Huilin Deng, Xin Huang, Pallavi Jayavanth, Ying Xiao, Jianlin Wu, Rui Jiao

**Affiliations:** 1 Department of Food Science and Engineering, Institute of Science and Technology, Jinan University, Guangzhou 510632, China; 13484879941@163.com (J.L.); zyqssifmail@163.com (Y.Z.); 15274066147@163.com (H.D.); huangxin11272020@163.com (X.H.); 2 State Key Laboratory of Quality Research in Chinese Medicine, Macau Institute for Applied Research in Medicine and Health, Macau University of Science and Technology, Taipa 999078, China; xxxuan_w@163.com (X.W.); jlwu@must.edu.mo (J.W.); 3 International School, Jinan University, 601 Huangpu Road, Guangzhou 510632, China; pallavijayavanth@gmail.com; 4 Faculty of Medicine, Macau University of Science and Technology, Taipa 999078, China; yxiao@must.edu.mo

**Keywords:** dark tea, theabrownin, insulin resistance, PI3K/Akt pathway, glucolipid metabolism

## Abstract

Dark tea has great potential in regulating glycolipid metabolism, and theabrownin (TB) is considered to be the characteristic and bioactive constituent of dark tea. This study evaluated the ability of TB1 (fermented for 7 days) and TB2 (fermented for 14 days) isolated from dark tea to reverse insulin resistance (IR) in HepG2 cells. The results indicated that TB significantly ameliorated oxidative stress by improving mitochondrial function. In addition, TB improved glycogen synthesis and glucose consumption, and inhibited gluconeogenesis and fatty acid synthesis, by regulating GSK3β (Glycogen synthase kinase 3β), G6Pase (Glucose-6-phosphatase), GCK (Glucokinase), PEPCK1 (Phosphoenolpyruvate carboxy kinase 1), SREBP-1C (sterol regulatory element-binding protein 1C), FASN (fatty acid synthase), and ACC (Acetyl-CoA carboxylase). Additionally, the results of Western blot and real-time PCR experiments demonstrated that TB modulated glucolipid metabolism through the IRS-1 (Insulin receptor substrate 1)/PI3K (phosphatidylinositol-3 kinase)/Akt (protein kinase B) signaling pathway. Treatment with the PI3K inhibitor demonstrated a favorable correlation between PI3K activation and TB action on glycolipid metabolism. Notably, we observed that TB2 had a greater effect on improving insulin resistance compared with TB1, which, due to its prolonged fermentation time, increased the degree of oxidative polymerization of TB.

## 1. Introduction

Nowadays, diabetes mellitus is recognized as a serious global health issue. According to the International Diabetes Federation, there will be 592 million diabetics worldwide by 2035. In China, it is estimated that 1 in 11 persons has diabetes, 90% of which is type 2 diabetes mellitus (T2DM). The overall prevalence of type 2 diabetes in China reached 14.92% from 2015 to 2019, while it was only 1.29% from 1980 to 1984 [1]. T2DM is one of the fastest growing metabolic diseases in the world, and insulin resistance (IR), a common metabolic disease that includes hyperglycemia and hyperlipidemia, can often be associated with it [2]. IR is a complex condition whereby the body fails to respond adequately to insulin, a hormone produced by the pancreas that is essential for life and regulating blood glucose levels [3]. Persistent hyperglycemia is known to elevate oxidative stress and induced IR in target tissues, thereby playing a significant role in the development of T2DM. Furthermore, impaired mitochondrial function caused by IR can lead to reducing insulin sensitivity and elevating blood glucose levels in a vicious cycle [4].

The liver is essential for the metabolism of nutrients. Maintaining metabolic homeostasis in the liver relies on the regulatory functions of two key metabolic hormones: insulin, which governs liver metabolism during fed states, and glucagon, which assumes this role during fasting states [5]. Furthermore, the PI3K/Akt pathway plays a crucial role in regulating glucose metabolism, with IRS-1 acting as a key regulator [6]. P-Akt regulates the activity of GSK3β and GYS (Glycogen synthase), ultimately promoting glycogen synthesis. Additionally, P-Akt blocks the activity of crucial gluconeogenesis-related enzymes including G6Pase and PEPCK1; thus, gluconeogenesis is suppressed [7]. In addition, the PI3K/Akt pathway regulates lipogenesis by inhibiting SREBP-2C (sterol regulatory element-binding protein 2C), which leads to increasing hepatic LDLR (low-density lipoprotein receptor) expression [8]. It is widely believed that high-glucose- and high-fat-induced IR is associated with high levels of reactive oxygen species (ROS), which lead to oxidative stress in the liver. In addition, excessive ROS can cause mitochondrial dysfunction, manifested as morphological changes and loss of function in mitochondria [9]. Consequently, reducing oxidative stress is effective in improving IR and preventing the progression of T2DM.

Long-term use of drugs that treat T2DM, such as metformin (MET), can lead to many side effects and drug resistance [10]. Besides, hundreds of millions of people with prediabetes are not recommended to take medications at first but make lifestyle changes instead. Therefore, it is particularly important to find functional foods to reverse insulin resistance and prediabetes without side effects [11].

Chinese dark tea has a long history and is popular for its unique aroma and taste [12]. There are several types of Chinese dark tea, including Pu’er tea from Yunnan, Liubao tea from Guangxi, Kangzhuan tea from Sichuan, Anhua black tea from Hunan, Qingzhuan tea from Hubei, and Fuzhuan tea from Shaanxi [13]. Dark tea differs from other teas in that it undergoes a microbial post-fermentation process, which produces special beneficial components. Research has shown that dark tea has hyperuricemic, hyperlipidemic, antidiabetic, antioxidative, and anti-obesity effects [14]. Previous studies have compared the weight loss effects of different teas and found that dark tea has a stronger effect on lowering blood sugar and lipids compared to green tea; however, the related mechanism has not been discussed [15].

Theabrownin (TB) is a water-soluble polymeric phenolic compound formed from various polyphenols, accounting for approximately 10–12% of the dry weight of mature dark tea, and may be composed of sugars, proteins, alkaloids, pigments, etc. [16,17,18]. During the fermentation process of dark tea, theaflavins are oxidized and polymerized to form thearubigins, then further formatted to theabrownin; at this stage, the concentration of theaflavins and thearubigins have decreased whereas the concentration of TB has significantly increased [19]. Studies have also shown that TB can effectively reduce skeletal muscle insulin resistance, accelerate fatty acid oxidation and mitochondrial biosynthesis, and inhibit the production of advanced glycation end products (AGEs) [20]. It can also significantly lower fasting blood sugar levels by promoting liver glycogen synthesis [21]. In addition, TB of dark tea promotes the growth of beneficial bacteria by changing the composition of intestinal flora, and the metabolites of these beneficial bacteria also have better anti-diabetic effects [22]; this indicates that TB is a particular and bioactive component of dark tea, which may be partially responsible for its hypolipidemic and hypoglycemic effects. However, the potential of TB with different fermentation times in ameliorating insulin resistance and its underlying mechanisms are still not clear.

Therefore, the present study is the first to (i) study the hypolipidemic and hypoglycemic effects of TB with different fermentation times (TB1: 7 days; TB2: 14 days); (ii) assess the impact of TB on oxidative stress and mitochondrial function; (iii) study the interaction of TB with the IRS-1/PI3K/Akt pathway and the proteins and genes related to glucolipid metabolism in insulin-resistant HepG2 cells.

## 2. Materials and Methods

### 2.1. Materials

Human HepG2 cells were bought from Fenghui Biotechnology Co., Ltd. (Changsha, China). In addition, this research also used the following chemicals: oleic acid and palmitic acid were purchased from Sigma Chemical Co., Ltd. (St. Louis, MO, USA); triglyceride (TG), total cholesterol (TC), HDL-cholesterol (HDL-C), LDL-cholesterol (LDL-C), glutathione (GSH), superoxide dismutase (SOD), malondialdehyde (MDA), and glucose detection kits were purchased from Nanjing Jiancheng Bio-Technology Co., Ltd. (Nanjing, China); glycogen assay kit was obtained from Boxbio Science & Technology Co., Ltd (Beijing, China); CCK-8 assay kit, ATP assay kit, lipophilic cationic probe 5,5′,6,6′-terachloro-1,1′,3,3′-tetraethyl-imidacarbocyanine iodide (JC-1) assay kit, and 2′,7′-dichlorofluorescein diacetate (DCFH-DA) were purchased from Biyuntian Biotechnology Co., Ltd. (Shanghai, China); bicinchoninic acid (BCA) assay kit was purchased from Applygen Technologies Co., Ltd. (Beijing, China).

A fluorescent D-glucose analog (2-NBDG) was acquired from Invitrogen Co., Ltd. (Calabasas, CA, USA). High-glucose Dulbecco’s modified Eagle’s medium (DMEM) and low-glucose Dulbecco’s modified Eagle’s medium (DMEM), RIPA buffer, and Trizol total RNA extraction kit were purchased from KeyGEN Bio TECH Co., Ltd. (Nanjing, China). Metformin (HPLC purity > 98%) was obtained from Solarbio Technology Co., Ltd. (Beijing, China). MitoSOX Red Mitochondrial Superoxide Indicator was acquired from Yeasen Bio Technologies Co., Ltd. (Shanghai, China). LY294002 (HPLC purity > 99.0%) was obtained from CSNpharm Co., Ltd. (Shanghai, China). Antibodies for HMGCR, SREBP-1C, SREBP-2C, ACC, P-ACC, LDLR, PCSK9, and β-Actin were acquired from Abcam (Cambridge, UK); P-IRS-1, IRS-1, Akt, P-Akt, PI3K, P-PI3K, FOXO1, P-FOXO1, GLUT2, GLUT4, G6Pase, GYS, P-GYS, GSK3β, P-GSK3β, GCK, PEPCK1, FASN, and GPAT1 were obtained from Zen-Bioscience Co., Ltd. (Chengdu, China). The secondary anti-rabbit IgG and anti-mouse IgG were obtained from Proteinch Biotech Co., Ltd. (Rockford, IL, USA). Methylene chloride, chloroform, ethyl acetate, and n-butanol were obtained from Anaqua Chemicals Supply Inc., Ltd. (Houston, TA, USA).

### 2.2. Dark Tea Samples Preparation

The first two pile-turning samples of dark tea were collected from Yunnan Province, China. The whole fermentation process took about 14 days [12]. Depending on the temperature of the tea pile and the manufacturer’s experience, tea mass was separated and showered with water, mixed, and restacked in the pile for about 7 days. In this work, re-piling and mixing were repeated two times. Samples from the upper layer of tea mass were selected.

### 2.3. TB Extraction

The extraction of TB was according to the reported literature with some modifications [23]. Dark tea’s level of oxidation deepens with increased fermentation time, increasing the yield of theabrownin. Theabrownin extracted from fermentation for one week was named TB1, and theabrownin extracted from fermentation for two weeks was named TB2. Their yields were 1.46% and 1.57%, respectively.

### 2.4. Ultraviolet—Visible (UV) and Fourier Transform Infrared (FT-IR) Spectroscopy

The UV–vis absorption spectra of the colloids were measured by using a UV—vis Spectrophotometer (Shimadzu, UV-2700, Kyoto, Japan). TB samples of 0.05 mg/mL were prepared. The spectra were recorded in the range of 200–800 nm with distilled water as the blank in a quartz cuvette. TB samples mixed with KBr were determined by FT-IR (IRAffinity-1S, SHIMADZU, Japan) within the wavenumber range of 4000–250 cm^−1^.

### 2.5. Cell Cultures

HepG2 cells were cultured in low-glucose (5.5 mM) DMEM supplemented with 10% fetal bovine serum and 1% Penicillin-Streptomycin at 37 °C, 5% CO_2_ atmosphere, and it was inoculated into 96-well plates (100 μL/Well, 5-well) at a density of 5 × 10^4^ cells/mL. To establish the IR model, HepG2 cells were cultured in high-glucose (25 mM) DMEM plus FFA (0.25, 0.5, 0.75, 1, 1.5 mM; OA (oleic acid):PA (palmitic acid) = 2:1) for 24 h, and the normal group incubated with low-glucose DMEM without FFA. Cell viability and glucose consumption of cells (measured in the following sections) were verified to determine that the IR model was successful.

### 2.6. CCK-8 Assay

At a density of 1 × 10^4^ cells/well, HepG2 cells were planted in 96-well plates and cultivated overnight. Cell viability was assessed using the CCK-8 assay after cells were treated with the specified doses of TB (50, 100, 150, 200 μg/mL) and metformin (15, 25, 35, and 45 μg/mL) for 24 h [24]. The absorbance was determined at 450 nm with an Infinite^®^ F50 ELISA reader (Tecan, Mannedorf, Switzerland). Then, the viability of cells treated with high-glucose DMEM plus FFA (OA:PA = 2:1, 0.5 mM) for 24 h in the presence of different TB (150 μg/mL) or MET (25 μg/mL) amounts was measured to confirm the safe dose of TB and MET in experiments.

### 2.7. Glucose Consumption Assay

HepG2 cells (1 × 10^5^ cells/mL) were planted in 96-well plates. After 24 h of treatment, the previous solution was discarded and changed with glucose medium containing 100 nmol/L insulin, then incubated for 3 h. Glucose consumption was detected with a glucose test kit (Jiancheng, China) and the OD values were obtained at 505 nm.

### 2.8. Glucose Uptake Assay

HepG2 cells (5 × 10^5^ cells/mL) were introduced in 6-well plates. With various interventions, HepG2 cells were treated with 0.1 mmol/L 2-NBDG and 100 nmol/L insulin at 37 °C for 3 h. Images were acquired at 470 nm (excitation) and 545 nm (emission) using a Leica inverted fluorescence microscope (Wetzlar, Württemberg, Germany), and the fluorescence intensity of 2-NBDG (reflecting the level of glucose uptake) was quantified by Image-J (Bethesda, MD, USA). Glycogen content in the cells was measured using a glycogen assay kit (Boxbio Science & Technology, Beijing, China).

### 2.9. Intracellular Lipid Profiles Test and Oil Red O (ORO) Staining

HepG2 cells were planted in a 6-well plate at 5 × 10^5^ cells/well and exposed to treatments for 24 h. The levels of TG, TC, HDL-C, and LDL-C levels were assessed using enzymatic test kits in IR-HepG2 cells. HepG2 cells were stained using the ORO method to assess the accumulation of neutral lipids and lipid droplet shape. After being cleaned with PBS, the cells were fixed for 10 min in 4% paraformaldehyde. After fixing, oil red staining solution was used for an hour at room temperature. The results were examined under a microscope after PBS washing (Optec instrument, Chongqing, China).

### 2.10. IR-HepG2 Cells Oxidative Stress Assay

HepG2 cells were seeded into 6-well plates at a confluence rate of 80% and cultivated for 24 h at 37 °C with 5% CO_2_. Following the GSH, MDA, and SOD kits’ instructions, each indication was measured. ATP concentration was assessed using an ATP kit. Mitochondrial membrane potential (MMP), cellular ROS, and mitochondrial ROS levels were analyzed according to previous methods and adjusted appropriately [25]. After 24 h of treatment, cells were incubated with JC-1 staining working solution, DCFH-DA (10 mM), or MitoSOX™ Red Mitochondria Superoxide indicator (5 μM) in dark conditions at 37 °C for 30 min. Behind the intervention, the cells were cleaned with PBS and observed under a Leica inverted fluorescent microscope (Wetzlar, Württemberg, Germany). The MMP value was the intensity ratio of red fluorescence to green fluorescence. Intracellular and mitochondrial ROS levels were shown by fluorescence intensity and quantified by Image-J (Bethesda, MD, USA).

### 2.11. Analysis of RT-PCR

The RNA in HepG2 cells was extracted using the Trizol Total RNA Extraction Kit (Meiji, Guangzhou, China); then, it was reverse-transcribed into cDNA using the PrimeScript RT reagent Kit (Accurate, Changsha, China) and stored at −80 °C. Real-time PCR was executed using SYBR green-based PCR Master Mix (Accurate, Changsha, China), utilizing a QuantstudioTM Real-Time PCR machine for quantitative analysis (Waltham, MA, USA). The primer sequences of the target genes are listed in Supplementary Materials Table S1.

### 2.12. Western Blot Assay

As previously described, the protein concentrations of regulators were assessed by Western blot analysis [26]. HepG2 cells (5 × 10^5^ cells/mL) were cultured in 6-well plates. After TB treatment, cells were collected by washing three times with PBS, lysed with RIPA buffer on ice for 20 min, then centrifuged at 12,000 rpm for 15 min to collect the supernatant. Using a BCA test kit (Biyuntian, Shanghai, China), protein concentration was measured. Proteins were separated by sodium dodecyl sulfate/polyacrylamide gel electrophoresis and then transferred to activated 0.22 m polyvinylidene fluoride (PVDF) membranes to determine the concentration of total proteins.

After blocking the PVDF membrane with skim milk for 1.5 h, the primary antibody was incubated at 4 °C overnight. The membrane was washed three times for ten minutes each with Tween 20 (TBST) in Tris-buffered saline, and then incubated with the secondary antibody for an hour at room temperature. ECL was added to the membrane to react for 1 min, then exposed, developed, and fixed. The relative intensity of the target protein band was analyzed using β-actin as an internal reference. In addition, HepG2 cells induced by high-glucose (25 mM) and high-fat (0.5 mM FFA) culture medium were treated with 10 nM LY294002 for 24 h; then, metformin (25 μg/mL), TB1 (150 μg/mL), and TB2 (150 μg/mL) were added for 24 h. The expression of related protein in the cells was analyzed according to the above method.

### 2.13. Statistical Analysis

All analyses were repeated three times. The data are presented as mean ± SD. Differences between the groups were evaluated by one-way analysis of variance (ANOVA) in GraphPad prism 8 (GraphPad Software Inc., San Diego, CA, USA). *p* < 0.05 indicates a significant difference, and *p* < 0.01 indicates an extremely significant difference.

## 3. Results

### 3.1. UV Spectrum and FT-IR of TB

**Figure 1:**
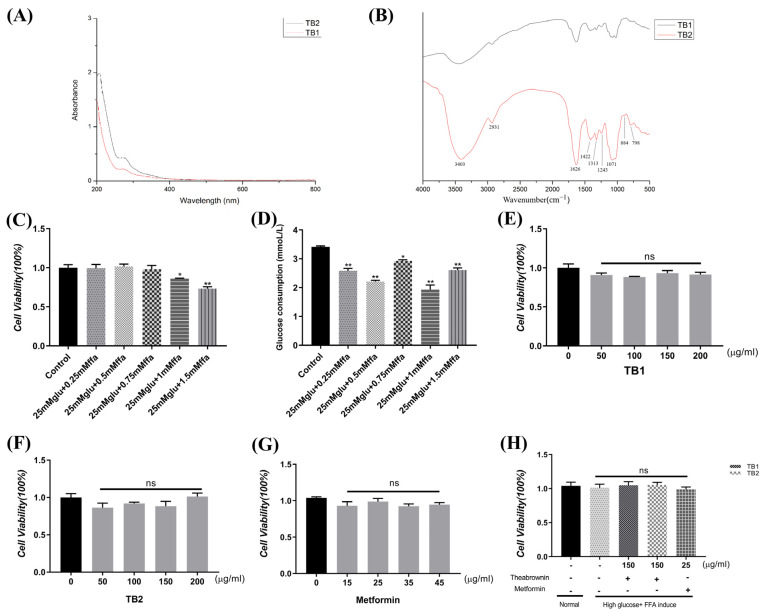
Preliminary characterization of TB, establishment of insulin resistance (IR) model and determination of cell viability by CCK8 assay. (**A**) UV spectrograms of TB1 and TB2 at 200–800 nm. (**B**) FT-IR spectra of TB1 and TB2. (**C**) Cell viability assay of HepG2 cells treated with high glucose (25 mmol/L) and different concentrations of FFA (0.25, 0.5, 0.75, 1, and 1.5 mmol/L). (**D**) Glucose consumption of IR-HepG2 cells by high glucose and different concentrations of FFA. (**E**–**G**) Cells were treated with increasing concentrations of TB (50–200 μg/mL), TB2 (50–200 μg/mL), and metformin (15–45 μg/mL) for 24 h. (**H**) Cells were treated with 25 mM glucose and 0.5 mM FFA for 24 h, and then handled with 150 μg/mL TB1 and TB2, and 25 μg/mL MET, for 24 h. CCK-8 kits were used to measure cell viability. All data are expressed as mean ± S.D. (*n* = 5) for each group. ns: no significance, * *p* < 0.05, ** *p* < 0.01 vs. control group.

UV–Vis spectroscopy can infer the molecular skeleton of compounds; judge the conjugation relationship between chromophores; and estimate the type, location, and number of substituents in the conjugation system. As shown in Figure 1A, all TB samples exhibited absorption peaks at 205 nm and 275 nm, which originated from the π–π* transitions of aromatic C=C in substituted benzenes or polyphenols [27]. The two absorption peaks in TB1 were relatively lower than those in TB2. Theabrownin was considered to be formed mainly by the polymerization of polyphenols [28]. TB1 was less fermented and the degree of phenol was lower than TB2, which may be the reason for its lower UV absorption.

The chemical bonds and functional groups of TB samples were further analyzed by FT-IR (Figure 1B). The broadly stretched intense peak at around 3403 cm^−1^ represents the stretching of the O-H. The small peaks observed at around 2931 cm^−1^ were attributed to the C-H stretching vibration [27]. The absorption peak occurring at 1626 cm^−1^ was attributed to C=O asymmetric stretching vibration of aromatic rings. The absorption band at 1422 cm^−1^ was the stretching vibration of aromatic C=C [28]. The peak at 1313 cm^−1^ was caused by the in-plane bending vibration of –OH. The absorption peaks at 1243 cm^−1^ and 1071 cm^−1^ were assigned to the C-OH. The other bands at 884 and 798 could be attributed to the substituents on the benzene rings [29]. These results suggested that TB samples were rich in hydroxyl and carboxylic groups and that they contained phenolic polymers [27]. In addition, absorption peaks in TB2 were stronger than in TB1, which may be due to the fact that the degree of polyphenol polymerization was deepened during fermentation.

### 3.2. Effect of TB on Cell Viability in IR-HepG2 Cells

In order to ensure safe doses for IR-related experiments, the effects of TB1, TB2, and MET on the viability of HepG2 cells were measured (Figure 1E–G). With the increase in TB concentration (50~200 μg/mL) and MET concentration (15~45 μg/mL), the viability of HepG2 cells did not change, elucidating that TB and MET in such concentrations had no cytotoxic effect on IR-HepG2 cells. Additionally, 25 mM glucose and various FFA concentrations were provided to HepG2 cells, and cell viability and glucose consumption were determined (Figure 1C,D). Based on these results, the final modeling conditions of 25 mM high glucose and 0.5 mM FFA, 150 μg/mL TB1 and TB2, and 25 μg/mL MET were selected for subsequent experiments.

### 3.3. Effect of TB on Glycometabolism in IR-HepG2 Cells

**Figure 2:**
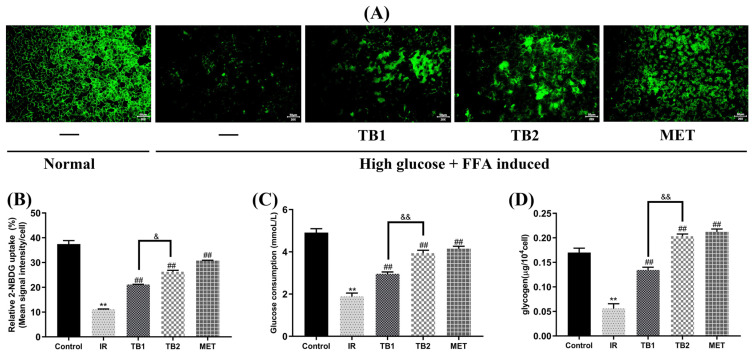
Effects of TB on glycometabolism in IR-HepG2 cells. (**A**) 2-NBDG tests for the uptake of glucose from IR-HepG2 cells. The values of glucose uptake were acquired from the intensity of the fluorescence pictures (50 μm, 20×) and (**B**) image analysis, (**C**) Glucose consumption, and (**D**) glycogen content. All data are expressed as mean ± S.D. (n = 5) for each group. ** *p* < 0.01 vs. control group; ## *p* < 0.01 vs. IR model group; & *p* < 0.05, && *p* < 0.01 vs. TB2 group.

To investigate the possible role of TB in high-glucose- and high-fat-induced IR, the effects of TB at 150 μg/mL on glycometabolism in IR-HepG2 cells were examined (Figure 2). Compared with the IR group, the results showed that TB1 increased glucose uptake by 47.72%, glucose consumption by 36.41%, and glycogen content by 58.44% (*p* < 0.01). TB2 increased glucose uptake by 57.95%, glucose consumption by 52.14%, and glycogen content by 72.50% (*p* < 0.01). It is important to note that the effect of TB2 on improving glucose metabolism is better than that of TB1 (*p* < 0.01), which is closer to the positive drug. These findings suggest that TB could enhance the metabolism of glucose in IR-HepG2 cells.

### 3.4. Effect of TB on Lipid Accumulation in IR-HepG2 Cells

**Figure 3:**
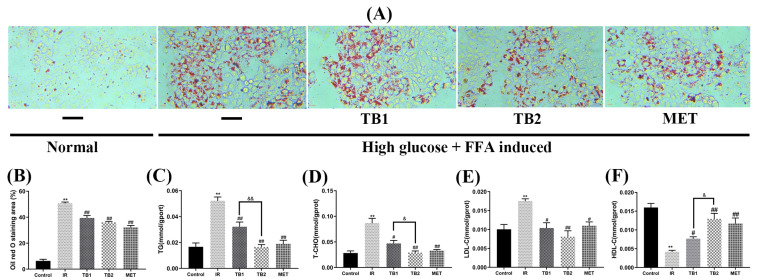
Effect of TB on lipid accumulation in IR-HepG2 cells. (**A**) Visual evaluation of lipid accumulation was captured using a microscope (200 μm, 20×): HepG2 cells were stimulated with high glucose (25 mM) and high fat (0.5 mM FFA) for 24 h, treated with TB and metformin for 24 h and stained with oil red. (**B**) Quantitative analysis by image, (**C**) TG, (**D**) TC, (**E**) LDL-C, and (**F**) HDL-C, Data are presented as the mean value ± SD (*n* = 5). ** *p* < 0.01 vs. control group; # *p* < 0.05, ## *p* < 0.01 vs. IR model group; & *p* < 0.05, && *p* < 0.01 vs. TB2 group.

Oil red staining was utilized to visualize the morphology and quantify the lipid accumulation of IR-HepG2 cells (Figure 3A). Intracellular lipid accumulation was inhibited after TB treatment (Figure 3B). Compared with the IR group, TB2 reduced TC by 66.10% (*p* < 0.01), reduced TG by 68.17% (*p* < 0.01), reduced LDL-C by 53.65% (*p* < 0.01), and increased HDL-C by 67.93% (*p* < 0.01). It can be seen that the effect of TB2 at 150 μg/mL is the same as that at 25 μg/mL MET (Figure 3C–F). It is further confirmed from the cellular level that TB2 has a considerable improvement effect on insulin resistance and lipid metabolism disorders.

### 3.5. Effect of TB on Oxidative Stress in IR-HepG2 Cells

**Figure 4:**
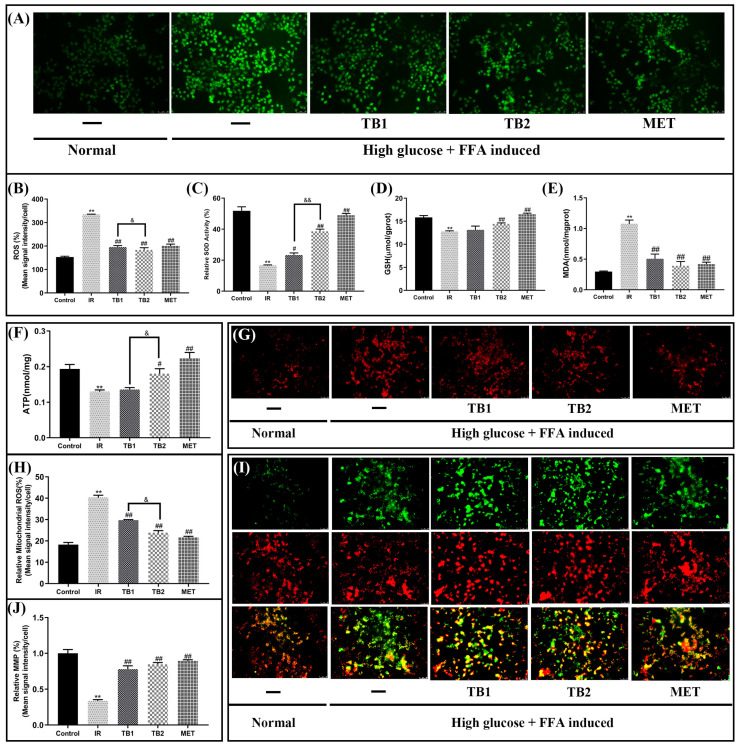
Effects of TB on mitochondrial function and oxidative stress of IR-HepG2 cells. (**A**,**B**) ROS levels in IR-HepG2 cells measured by microplate fluorometry (50 μm, 20×). (**C**–**E**) SOD, GSH, and MDA levels in IR-HepG2 cells. (**F**) ATP contents. (**G**,**I**) Mitochondrial ROS. (**H**,**J**) MMP levels are shown as the ratio of red/green using fluorescence microscopy (50 μm, 20×). Data are presented as the mean value ± SD (*n* = 5). ** *p* < 0.01 vs. control group; # *p* < 0.05, ## *p* < 0.01 vs. IR model group; & *p* < 0.05, && *p* < 0.01 vs. TB2 group.

Oxidative-stress-induced mitochondrial dysfunction plays a significant role in the evolution of T2DM [30]. Compared with the IR group, 150 μg/mL TB2 treatment increased the level of SOD in the cell by 57.09% (*p* < 0.01), GSH increased by 11.65% (*p* < 0.01), and ATP increased by 26.1% (*p* < 0.05). The treatment of 150 μg/mL TB1 increased the level of SOD in the cell by 28.09% (*p* < 0.05), the level of GSH increased by 3.09%, and the level of ATP increased by 4.55% (Figure 4C,D,F). Compared with the IR group, TB1, TB2, and MET interventions reduced the content of MDA by 52.89%, 63.61%, and 61.03%, respectively (*p* < 0.01) (Figure 4E). According to Figure 4G, the mitochondrial ROS of the IR-HepG2 group were higher than that of the normal group (*p* < 0.01); TB1 and TB2 treatments decreased mitochondrial ROS production by 26.24% and 40.88%, respectively. Obviously, TB2 significantly inhibited ROS production (*p* < 0.05). When examining MMP values in IR-HepG2 cells, a similar trend to ATP content was observed (Figure 4I,J). This means that TB2 is more capable of reducing mitochondrial oxidative stress than TB1 (*p* < 0.01), maintaining normal glucolipid metabolism and energy supply, and improving mitochondrial function.

### 3.6. Effects of TB on Key Proteins and Gene Expressions of Glucose Metabolism in IR-HepG2 Cells

**Figure 5:**
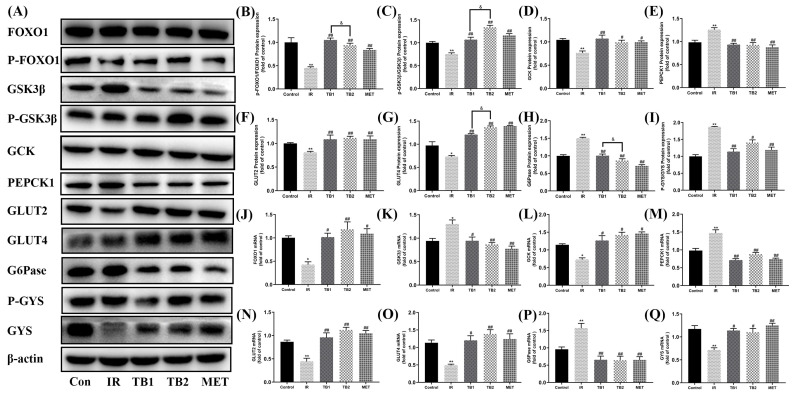
TB treatment ameliorated expression of genes and proteins related to glucose metabolism. (**A**) Representative Western blot results of IR-HepG2 cells glycometabolism markers including FOXO1, P-FOXO1, GSK3β, P-GSK3β, GCK, PEPCK1, GLUT2, GLUT4, G6Pase, P-GYS, and GYS. (**B**–**I**) Relative values of P-FOXO1/FOXO1, P-GSK3β/GSK3β, GCK, PEPCK1, GLUT2, GLUT4, G6PC, and P-GYS/GYS proteins. (**J**–**Q**) Relative values of FOXO1, GSK3β, GCK, PEPCK1, GLUT2, GLUT4, G6Pase, and GYS mRNA levels. Data are expressed as mean ± S.D. (*n* = 3). * *p* < 0.05, ** *p* < 0.01 vs. control group; # *p* < 0.05, ## *p* < 0.01 vs. IR model group; & *p* < 0.05 vs. TB2 group.

To further study the effect of TB on the glycometabolism of IR-HepG2 cells, the expression levels of key proteins were determined by WB and RT-PCR. Compared with the normal group, the glycogen content of the IR group was significantly decreased; the ratio of *p*-GYS/GYS was increased (Figure 5I) (*p* < 0.01), with the ratio of *p*-GSK3β/GSK3β (Figure 5C) (*p* < 0.01); and GCK (Figure 5D) (*p* < 0.01) was decreased, indicating that the synthesis of glycogen in the model cells was significantly inhibited, unable to utilize glucose. This trend can be reversed after TB intervention, with TB2 showing a better improvement effect than TB1 (*p* < 0.05). Moreover, TB promoted the phosphorylation of FOXO1 (Forkhead transcription factor1) (Figure 5B) and downregulated the protein and mRNA levels of G6Pase (Figure 5H,P) and PEPCK1 (Figure 5E,M), inhibiting liver gluconeogenesis and reducing glucose production. We found that TB2 stimulation significantly increased FOXO1 phosphorylation and decreased G6Pase expression compared with TB1 (*p* < 0.05). GLUT2 and GLUT4 are key enzymes in the hepatic glucose transport process [31]. Under insulin resistance conditions, the protein expressions of GLUT2 and GLUT4 on the cell membrane of IR-HepG2 cells were decreased (Figure 5F,G) (*p* < 0.05), impairing glucose utilization. Compared with the control group, the mRNA expression levels of GLUT2 and GLUT4 were increased in the TB-treated group (Figure 5N,O) (*p* < 0.05). These results indicate that TB improved enzymes’ expression to promote glucose uptake and improve glucose utilization, while TB2 presented better properties for improving glucose metabolism than TB1 (*p* < 0.05).

### 3.7. Effects of TB on Key Proteins and Gene Expressions of Lipid Metabolism in IR-HepG2 Cells

**Figure 6:**
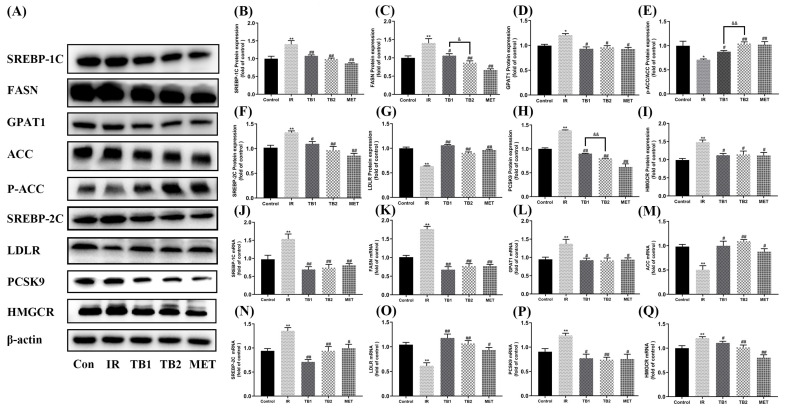
TB treatment ameliorated expression of genes and proteins associated with lipid metabolism. (**A**) Representative Western blot results of IR-HepG2 cells lipid metabolism markers including SREBP-1C, FASN, GPAT1, ACC, P-ACC, SREBP-2C, LDLR, PCSK9, and HMGCR. (**B**–**I**) Relative values of SREBP-1C, FASN, GPAT1, P-ACC/ACC, SREBP-2C, LDLR, PCSK9, and HMGCR proteins. (**J**–**Q**) Relative values of SREBP1-C, FASN, GPAT1, ACC, LDLR, PCSK9, and HMGCR mRNA levels. Data are expressed as mean ± S.D. (*n* = 3). * *p* < 0.05, ** *p* < 0.01 vs. control group; # *p* < 0.05, ## *p* < 0.01 vs. IR model group; & *p* < 0.05, && *p* < 0.01 vs. TB2 group.

The insulin-dependent PI3K/Akt pathway plays a crucial role in activating SREBP, an essential transcription factor in regulating fatty acid and cholesterol synthesis pathways [32]. TB intervention significantly decreased the protein and gene expression levels of SREBP-1C (Figure 6B,J) (*p* < 0.01), FASN (Figure 6C,K) (*p* < 0.01), and GPAT1 (Figure 6D,L) (*p* < 0.01), while increasing the expression of P-ACC/ACC (Figure 6E) (*p* < 0.01), leading to a reduction in hepatic triglycerides. Notably, TB2 stimulation significantly decreased the expression of FASN and increased ACC phosphorylation compared with the TB1 group (*p* < 0.05). Furthermore, TB was found to downregulate SREBP-2C (Figure 6F,N) and HMGCR (Figure 6I,Q) protein and mRNA levels (*p* < 0.05), indicating that it can inhibit cholesterol synthesis in IR-HepG2 cells. TB also upregulated the mRNA and protein levels of LDLR (Figure 6G,O) (*p* < 0.05) while inhibiting the protein and mRNA expressions of PCSK9 (Proprotein convertase subtilisin/kexin type 9) (Figure 6H,P) (*p* < 0.05) to promote the clearance of plasma LDL-C and decrease the cholesterol level in the liver. As expected, TB2 was found to have a greater inhibitory effect on PCSK9 levels compared to TB1 (*p* < 0.05), suggesting a better lipid-lowering activity of TB2.

### 3.8. Effects of TB on Ameliorating High-Glucose- and High-Fat-Induced IR through PI3K/Akt Pathway in HepG2 Cells

**Figure 7:**
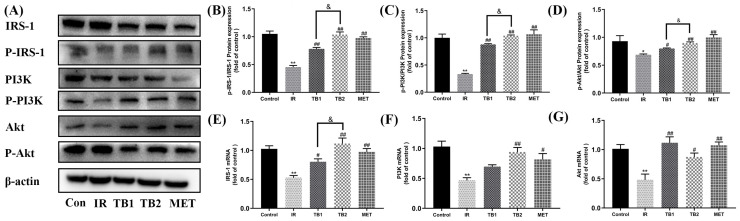
Effects of TB on the IRS-1/PI3K/Akt pathway in IR-HepG2 cells. (**A**) Western blot analysis was used to determine the expression levels of IRS-1, P-IRS-1, PI3K, P-PI3K, Akt, and P-Akt in IR-HepG2 cells. (**B**) P-IRS-1/P-IRS-1 expression. (**C**) P-PI3K/PI3K expression. (**D**) P-Akt/Akt expression. (**E**) IRS-1 mRNA. (**F**) PI3K mRNA. (**G**) Akt mRNA. Data are expressed as mean ± S.D. (*n* = 3). * *p* < 0.05, ** *p* < 0.01 vs. control group; # *p* < 0.05, ## *p* < 0.01 vs. IR model group; & *p* < 0.05 vs. TB2 group.

To further study the mechanism underlying the impact of TB on glucolipid metabolism in IR-HepG2 cells, the levels of IRS-1, PI3K, and Akt in IR-HepG2 cells were evaluated by Western blot and RT-PCR. TB1 and TB2 interventions increased P-IRS-1/IRS-1 by 58.3% (*p* < 0.01) and 32.7% (*p* < 0.01) (Figure 7B), P-PI3K/PI3K by 54.6% (*p* < 0.01) and 70.6% (*p* < 0.01) (Figure 7C), and P-Akt/Akt by 11.2% (*p* < 0.05) and 21.0% (*p* < 0.01) (Figure 7D), respectively. Compared with TB1, TB2 has a more obvious positive effect on the IRS-1/PI3K/Akt pathway (*p* < 0.05). Our detection of the mRNA levels of these genes has also been similarly validated (Figure 7E–G).

**Figure 8:**
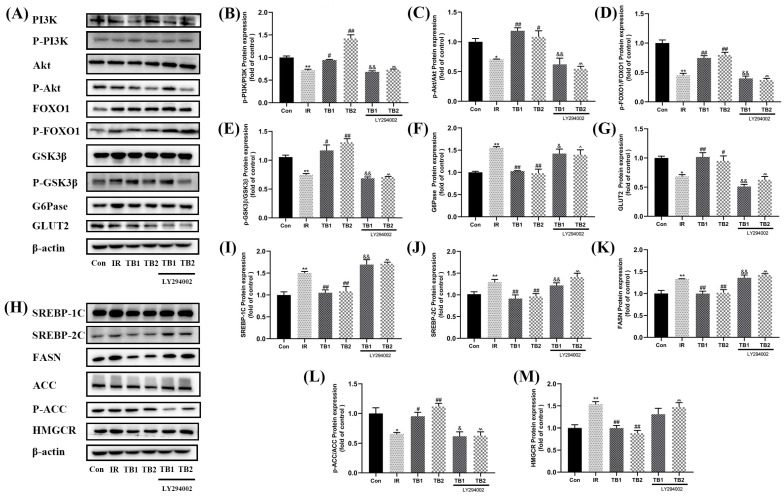
TB ameliorates high-glucose and high-fat-induced IR through inhibition of PI3K/Akt signaling pathway. (**A**) The expression levels of PI3K, P-PI3K, Akt, P-Akt, FOXO1, P-FOXO1, GSK3β, P-GSK3β, G6Pase, and GLUT2 in IR-HepG2 cells were detected by Western blot. (**B**–**G**) Relative values of P-PI3K/PI3K, P-Akt/Akt, P-FOXO1/FOXO1, P-GSK3β/GSK3β, G6Pase, and GLUT2 proteins. (**H**) The expression levels of SREBP-1C, SREBP-2C, FASN, ACC, P-ACC, and HMGCR in IR-HepG2 cells were detected by Western blot. (**I**–**M**) Relative values of SREBP-1C, SREBP-2C, FASN, P-ACC/ACC, and HMGCR proteins. Data are expressed as mean ± S.D. (*n* = 3). * *p* < 0.05, ** *p* < 0.01 vs. control group; # *p* < 0.05, ## *p* < 0.01 vs. IR model group; & *p* < 0.05, && *p* < 0.01 vs. IR-TB1 group; ^ *p* < 0.05, ^^ *p* < 0.01 vs. TB2 group.

HepG2 cells were pretreated with a particular PI3K inhibitor (LY294002) for 24 h in order to clarify if the PI3K pathway is implicated in the action of TB on high-glucose- and high-fat-induced IR. The findings demonstrate that LY294002 (10 nM) successfully inhibited PI3K phosphorylation and prevented *p*-IRS-1 from being expressed more highly in IR-HepG2 cells. Furthermore, inhibition of PI3K further reduced the expression of P-Akt compared with TB treatment, indicating that TB may exert its impact on high-glucose- and high-fat-induced IR through the PI3K/Akt pathway (Figure 8).

The PI3K/Akt pathway was then further investigated to determine how TB regulates high-glucose- and high-fat-induced IR. As shown in Figure 8B–G, the effects of TB on the phosphorylation of GSK3β and FOXO1, two Akt substrates, were inhibited when LY294002 was present. The protein expression levels of G6Pase and GLUT2 compared with the TB intervention group also significantly increased (*p* < 0.05), suggesting that TB’s effects on glucose metabolism are PI3K-dependent. In addition, according to Figure 8H–M, compared with the TB treatment group, the combined use of LY294002 and TB increased the expression levels of SREBP-1C and SREBP-2C (Akt lipid metabolism substrates), and the expression levels of their downstream targets FASN, ACC, and HMGCR also increased (*p* < 0.05); this suggests that the role of TB in preventing lipid metabolic disorders is PI3K-dependent. Overall, this work provides information about the underlying mechanism of TB’s beneficial effect on high-glucose- and high-fat-induced IR in HepG2 cells. We found that the expression of PI3K/Akt pathway was stronger in TB2 treatment than TB1, which may be the reason why TB2 regulates glucolipid metabolism better than TB1.

## 4. Discussion

IR is deeply associated with the progression of T2DM, which limits insulin’s ability to facilitate glucose uptake and promotes lipid accumulation, ultimately resulting in hyperglycemia [33]. Hyperglycemia, in turn, triggers oxidative stress and exacerbates damage to peripheral tissues, leading to the emergence of metabolic syndrome and its associated complications, such as neuropathy, nephropathy, and retinopathy. Hence, it is crucial to inhibit oxidative stress induced by high glucose and high fat levels to prevent IR and mitigate the complications of T2DM. Dark tea has the highest concentration of theabrownin (TB) of all tea kinds since it is produced by post-fermenting fresh camellia leaves [34]. The distinctive bioactivity of dark tea in comparison to other teas is thought to be mostly attributed to TB. So, it seems logical to hypothesize that TB may enhance glycolipid metabolism and show promise as a therapeutic drug for the management of type 2 diabetes and obesity [16]. TB1 (7 days) and TB2 (14 days) were extracted from dark tea according to different fermentation times. In the present study, TB1 and TB2 were found to modulate the IR HepG2 cell model by inhibiting the expression of IRS-1 and activating the PI3K/Akt pathway, thereby regulating the activity of enzymes involved in lipid and glucose metabolism. On the other hand, TB1 and TB2 improve insulin resistance by attenuating oxidative stress. Importantly, TB2 was more effective than TB1 in mitigating insulin resistance in HepG2 cells. TB2 has a better hypoglycemic and lipid lowering effect than TB1, which may be due to the increase in fermentation time and the increase in the degree of oxidation and polymerization of tea polyphenols. Prolonged fermentation may result in the generation of more theabrownin, thereby increasing the content of these bioactive compounds in the tea leaves.

The liver, as a pivotal metabolic organ, plays a crucial role in regulating the body’s energy metabolism. It serves as a connection point, connecting different tissues such as skeletal muscle and adipose tissue, and actively contributes to maintaining glucose homeostasis in the human body. The liver and its surrounding tissues are the primary sites of insulin resistance. There are various issues with related research that are based on human experimentation, including complexity, length of time, and significant disparities. Therefore, conducting in vitro cell tests is essential. Hepatic embryonal tumor cells called HepG2 are produced from humans, and their phenotype is extremely similar to that of hepatocytes. The number of insulin receptors on the surface of HepG2 cells dropped in response to high glycolipid concentrations, and the extent of the loss was positively linked with glycolipid concentration and the action time. HepG2 cells are, therefore, the best cells to use for researching the causes of insulin resistance and the mechanisms through which hypoglycemic drugs work [35].

We assessed the effect of TB on glycometabolism. Glucose in the blood can enter the liver cells, where it is decomposed and utilized [36]. Under conditions of insulin resistance, the ability of liver cells to reply to GLUT is weakened, resulting in decreased glucose uptake, which results in increased blood sugar levels [31]. Our research shows that TB can reverse the downregulation of GLUT2 and GLUT4 expression caused by high glucose and high fat, causing a rise in the rate of glucose uptake by hepatocytes. IR is known to reduce glucose absorption and consumption, decreasing the liver’s ability to use glucose (Figure 2) [37]. The process by which the liver breaks down non-carbohydrates to produce glucose is called gluconeogenesis [38]. Two rate-limiting enzymes that stimulate gluconeogenesis are PEPCK1 and G6Pase [39]. These two enzymes’ upregulation is also very closely related to insulin resistance. To increase the synthesis of glucose when fasting, the transcription factor FOXO1 interacts with other regulators to activate both G6Pase and PEPCK [32]. Glycogen production and breakdown are crucial steps in the body’s maintenance of glucose homeostasis [40]. The essential hepatocyte enzymes GSK3β and GYS are involved in the formation of glycogen [41]. In addition, GCK enzymes catalyze the glycolytic pathway’s first step, the conversion of glucose to glucose-6-phosphate [42]. The activity of the GCK enzyme is regulated by insulin, and the secretion of insulin will promote the activity of the GCK enzyme, thereby promoting the utilization and storage of glucose. Findings demonstrated that TB enhanced the expression of GCK enzymes (Figure 5D), thereby promoting glycolysis and glycogen synthesis. When considering all the results, it appears that TB has a dual mechanism of action in regulating glucose and glycogen metabolism. On one hand, TB increases the phosphorylation of AKT, leading to the inhibition of GSK3β activity and subsequent glycogen synthesis via increased activity of GYS. On the other hand, TB-induced *p*-AKT agonism also inhibits the expression of FOXO1, leading to decreased activity of PEPCK1 and G6Pase, and subsequently inhibiting glucose production. Notably, TB2 significantly restored the IR-induced decrease in glucose metabolism, which was not significantly different from metformin, which may be due to the enhanced TB activity caused by prolonged fermentation. Previous research has also suggested that dark tea anti-obesity may be achieved by regulating the IR signaling pathway and the PI3K/Akt/GLUT4 pathway [31].

In addition to modulating glucose metabolism, TB treatment can also reduce hepatic lipid accumulation [23]. Previous studies have established that dark tea can support a healthy gut microbiome and possesses greater potential than other teas in regulating lipid metabolism. These findings are consistent with those of other researchers [43]. We also found that TB extracted from dark tea can effectively intervene in treating IR-HepG2 cells, and TB2 exhibited better lipid-lowering activity compared to TB1 (Figure 3). In lipid metabolism, FASN and GPAT1 are key enzymes for lipid synthesis [40]. Our results indicated that TB reduced the expression of FASN and GPAT1 in IR-HepG2 cells and inhibited lipid synthesis from the source (Figure 6C,D). Additionally, ACC is a key regulator of fatty acid synthesis and oxidation pathways. It has been reported that SREBP-1C directly phosphorylates ACC, thereby reducing fatty acids in mitochondria and reducing the rate of fatty acid oxidation [44]. Furthermore, Akt interacted with SREBP-1C and directly stimulated Ser372 phosphorylation, leading to reduced SREBP-1C cleavage and nuclear translocation, and ultimately attenuated the expression of P-ACC target genes in IR-HepG2 cells, thereby reducing adipogenesis and lipid accumulation [45]. Other experiments also reported that TB can increase LDLR protein levels and that PCSK9 can bind to LDLR as a molecular chaperone, resulting in LDLR degradation [23]. In our study, the results suggest that this may be because TB can increase LDLR by downregulating PCSK9 (Figure 6G,H). In summary, we found that TB increased PI3K activation; increased Akt and ACC phosphorylation; and simultaneously decreased the levels of HMGCR and SREBP-2C to reduce cholesterol synthesis, and decreased PCSK9 nucleoprotein levels and increased LDLR protein levels to promote the elimination of LDL cholesterol (Figure 6).

We examined its impact on the IRS-1/PI3K/Akt pathway to better comprehend how TB penetrates cells and activates nuclear transcription factors (Figure 7). The IRS-1/PI3K/Akt pathway has been reported to be critical in regulating insulin sensitivity and metabolism [46]. Akt is considered to be a key regulator involved in glucose metabolism. GSK3 is inhibited by being phosphorylated by Akt, which then dephosphorylates and activates glycogen synthase to speed up glycogen production [47]. After activation of Akt kinase, glucose uptake is promoted by the glucose transporter GLUT4 and transported to the cell membrane [46]. In addition, Akt activation can inhibit the expression of PEPCK and G6Pase, and inhibit gluconeogenesis through FOXO1 phosphorylation [48]. Fuzhuan tea extract has reportedly been shown to reduce T2DM via altering the gut microbiota, regulating gut metabolites, and activating the IRS-1/PI3K/Akt pathway [49]. Consistently, our findings demonstrate that TB activates the insulin receptor and significantly increases the expression of P-IRS-1/IRS-1 (Figure 7B). Both TB1 and TB2 significantly enhanced PI3K and P-Akt/Akt ratios (Figure 7C,D), suggesting that TB’s improvement of high-glucose and high-fat-induced insulin resistance may be achieved by activating insulin-related cell signaling. Meanwhile, TB not only significantly decreased P-AKT/AKT, P-GSK3β/GSK3β, P-FOXO1/FOXO1, and P-ACC/ACC ratios in cells pretreated with LY294002 compared with normal cells without inhibitors but also decreased the expression of SREBP, FASN, and HMGCR, suggesting that the effects of TB on the regulation of glucolipid homeostasis are PI3K-dependent (Figure 8).

Oxidative stress is the common basis of diabetes and cardiovascular disease [50]. Elevated oxidative stress may be a major detrimental factor for insulin resistance, impaired mitochondrial function, abnormal energy metabolism, impaired insulin sensitivity, and dyslipidemia [51]. The combination of high blood sugar and high FFAs leads to high production of ROS. Recent research has shown that Akt inhibition caused by ROS is a typical cause of IR pathophysiology in T2DM [52]. Mitochondria regulate various metabolic processes and eliminate excess ROS production in the body caused by hyperglycemia [53]. Oxidative-stress-induced mitochondrial dysfunction in hepatocytes is closely related to insulin resistance [51]. SOD, MDA, and GSH are the key indicators to evaluate the degree of oxidative stress in the human body. SOD scavenges free radicals, MDA is a marker of oxidative stress, and GSH can reduce the damage caused by oxidative stress to the human body [54]. Therefore, studying the effects of TB on improving oxidative stress and mitochondrial function can further explore its mechanism of ameliorating insulin resistance. In our study, TB intervention led to a significant decrease in cellular ROS fluorescence intensity and MDA levels (Figure 4A,E), and an increase in SOD activity and GSH content (Figure 4C,D). Our results indicate that TB2 is more effective than TB1 in improving oxidative stress, and its activity is more similar to that of metformin. We also confirmed that IR-HepG2 cells developed oxidative stress and resulted in elevated mitochondrial ROS (Figure 4G), which can lead to mitochondrial dysfunction. Mitochondrial dysfunction will seriously impair ATP production; further, it will affect the MMP, leading to the utilization of blood sugar and causing hyperglycemia [55]. The study of TB’s effect on mitochondrial ATP and MMP levels proved the above concepts, showing that TB2 can increase ATP production (Figure 4F) and MMP (Figure 4I), and decrease oxidation stress in IR-HepG2 cells. This finding suggested that TB2 may have a favorable protective impact on insulin resistance by improving oxidative-stress-induced mitochondrial dysfunction. Furthermore, the results demonstrated that prolonging the fermentation time can increase the antioxidant activity of TB, potentially enhancing its effectiveness in combating insulin resistance. Previous studies have shown that Liubao tea (LBT) and Pu’er tea (PET) have better glycosidase inhibitory activity, hypoglycemia activity in vivo, and alleviate insulin resistance, while green brick tea (QBT) and Fuzhuan tea (FBT) have better free radical scavenging activity, which may be related to their different phytochemical composition. Therefore, dark tea is a promising option for anti-diabetic foods, and LBT and PET may be excellent naturally occurring sources of agricultural products with anti-diabetic properties [56].

## 5. Conclusions

**Figure 9:**
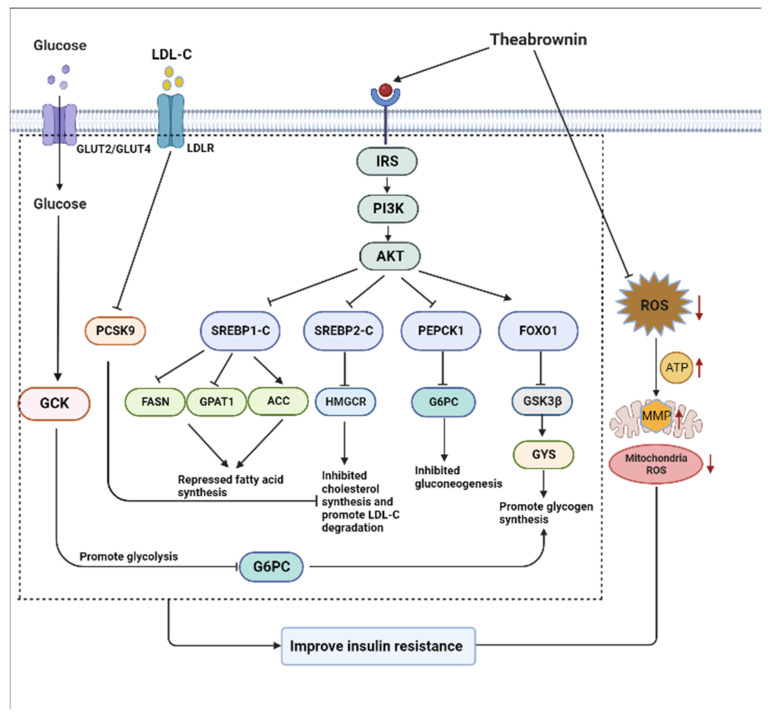
Potential cellular mechanism of action of theabrownin (TB) through activation of the IRS-1/PI3K/Akt pathway and downstream hepatic regulators in an insulin-resistant (IR) HepG2 cell model (“→” indicate promotion, “┨” indicate suppression), (Created with BioRender.com).

In conclusion, our findings strongly indicate that TB2 is more effective in regulating glucolipid metabolism, as well as improving oxidative stress and mitochondrial function, compared with TB1 in insulin-resistant HepG2 cells (Figure 9). Our findings also support that the effects of TB on insulin resistance are mainly related to fermentation time, and TB2 treatment with 14 days of fermentation is more effective than TB1 treatment with 7 days of fermentation. The cellular mechanism of TB action mainly refers to the activation of the IRS-1/PI3K/Akt pathway, followed by the regulation of the network among important transcription factors including SREBP-1C, P-FOXO1, FOXO1, and SREBP-2C. By regulating the expression of these transcription factors, TB further attenuated gluconeogenesis by inactivating G6Pase and PEPCK1, reduced adipogenesis by reducing FASN levels and phosphorylating ACC, and reduced cholesterol production by downregulating HMGCR. In addition, TB can reduce cellular and mitochondrial ROS levels, restore ATP production, and maintain MMP. The findings of this study serve as a useful reference for forthcoming clinical trials, presenting TB2 as a promising drug candidate in the effort to alleviate the global burden of insulin resistance and T2DM. However, further research on the therapeutic effect of TB on T2DM is needed by establishing T2MD mouse models and human clinical studies.

## Data Availability

Data will be made available on request.

## Supplementary Materials

The following supporting information can be downloaded at: https://www.mdpi.com/article/10.3390/nu15183862/s1, Table S1. Primers Used for Real-Time Quantitative PCR (PDF).
