# Supplementary material for: Theabrownin from Dark Tea Ameliorates Insulin Resistance via Attenuating Oxidative Stress and Modulating IRS-1/PI3K/Akt Pathway in HepG2 Cells
Source: Nutrients. 2023 Sep 5;15(18):3862. doi: 10.3390/nu15183862 (PMC10536292; doi:10.3390/nu15183862)
Supplement: Supplementary file 1 [file nutrients-15-03862-s001.zip › nutrients-2588175-supplementary.pdf]

Supplementary material

Theabrownin from Dark Tea Ameliorates Insulin Resistance via Attenuating Oxidative Stress and Modulating IRS-1/PI3K/Akt Pathway in HepG2 Cells

Jia Liu [1], Xuan Wang [2], Yuanqin Zhu [1], Huilin Deng [1], Xin Huang [1], Pallavi Jayavanth [3], Ying Xiao [4], Jianlin Wu [2] and Rui Jiao [1,*]

1 Department of Food Science and Engineering, Institute of Science and Technology, Jinan University, Guangzhou 510632, China; 13484879941@163.com (J.L.); zyqssifmail@163.com (Y.Z.); 15274066147@163.com (H.D.); huangxin11272020@163.com (X.H.)

2 State Key Laboratory of Quality Research in Chinese Medicine, Macau Institute for Applied Research in Medicine and Health, Macau University of Science and Technology, Taipa 999078, China; xxxuan_w@163.com (X.W.); jlwu@must.edu.mo (J.W.)

3 International School, Jinan University, Guangzhou 510632, China; pallavijayavanth@gmail.com

4 Faculty of Medicine, Macau University of Science and Technology, Taipa 999078, China;
yxiao@must.edu.mo

* Correspondence: tjiaorui@jnu.edu.cn

Table 1. Primers Used for Real-Time Quantitative PCR

Gene	Forward primer	Reverse primer
IRS-1	ACAAACGCTTCTTCGTACTGC	AGTCAGCCCGCTTGTTGATG
PI3K	TACAATACGGTGTGGAGTATGGA	TCATTGGCTTAGGTGGCTTTG
Akt	AGGCACGGGCTAAAGTGAC	CTGTGTGAGCGACTTCATCCT
GLUT-2	GCCTGGTTCCTATGTATATCGGT	GCCACAGATCATAATTGCCCAAG
GLUT-4	ATCCTTGGACGATTCCTCATTGG	CAGGTGAGTGGGAGCAATCT
GSK3β	GGCAGCATGAAAGTTAGCAGA	GGCGACCAGTTCTCCTGAATC
GYS	TGAAGTTGCTTGGGAAGTGAC	AGGTTCACACTGTTCCACCTG
FOXOI	TCGTCATAATCTGTCCCTACACA	CGGCTTCGGCTCTTAGCAAA
PEPCK1	CTGCTGGTCCCTCTAGTCTAT	CCAGGTATTTGCCGTTGTAG
G6Pase	GCCTCATCTATTGGACCCTCTTT	GCTATCTTCTGGCCATTTCCCA
GCK	TGCTACTACGAAGACCATCAGT	CCACTCGGTATTGACGCACA
GPAT1	GATGTAAGCACACAAGTGAGGA	TCCGACTCATTAGGCTTTCTTC
FASN	GTGTGGACATGGTCACAGATG	GACCGCTTGGGTAATCCATA
SREBP-2C	AACGGTCATTCACCCAGGTC	GGCTGAAGAATAGGAGTTGCC
LDLR	CAAAGTCTGCAACATGGCTAGAGA	GTTGTCCAAGCATTCGTTGGTC
ACC	CAACTTTGTGCCCACGGTTA	TTTGTCAGGAAGAGGCGGAT
SREBP1-C	GCTAGCTAGATGACCCTGCAC	GCAGCAGCAAGATTTGCCTA
PCSK9	CCTGGAGCGGATTACCCCT	CTGTATGCTGGTGTCTAGGAGA
HMGCR	CTTGTGTGTCCTTGGTATTAGAGCTT	GCTGAGCTGCCAAATTGGA
β-actin	TGGCACCCAGCACAATGAA	CTAAGTCATAGTCCGCCTAGAAGCA
